# Apparent Exercise of Volition during Anæsthesia Complete in All Other Respects

**Published:** 1868-12

**Authors:** W. H. Shepherd

**Affiliations:** Carter’s Bridge, Va.


CARTER'S BRIDGE, VA., 1868.

APPARENT EXERCISE OF VOLITION DURING ANÆSTHESIA COMPLETE IN ALL OTHER RESPECTS.—Called, March 3d, 1868, to M——, æt. 10 years; female, nervous temperament fully marked; suffering with odontalgia; was very unwilling to allow extraction of the diseased tooth, and closed the jaws so strenuously as to successfully resist every effort to part them. At the solicitation of the parents, I subjected her to the influence of chloroform, during the administration of which, she had to be forcibly held upon the couch, so great was her repugnance to extraction, even when assured it would be painless. I repeated my efforts to separate the jaws, while anæsthesia was yet imperfect, but opposing volition was still exercised and baffled the attempts. During this period she opened the mouth and permitted an examination of the tooth, under the assurance that I would not attempt its extraction. When every other evidence of full and complete anæsthesia was present, I still found the jaws so strongly held together as to necessitate leverage in opening the mouth for the admission of the extracting forceps. The resistance was not such as we find in tonic spasm, but appeared to be the result of voluntary effort, and never yielded, though the anæsthetic was used until the condition of the pupils and pulse forbade its further employment. There was not the least sign of any spasm or rigidity of any other muscles of the system; but, on the contrary, the usual relaxation.

W. H. SHEPHERD, M.D.

—*Richmond and Louisville Medical Journal.*

